# An atlas of small RNAs from potato

**DOI:** 10.1002/pld3.466

**Published:** 2022-12-14

**Authors:** Patricia Baldrich, Alexander Liu, Blake C. Meyers, Vincent N. Fondong

**Affiliations:** ^1^ Donald Danforth Plant Science Center St. Louis Missouri USA; ^2^ Division of Plant Science & Technology University of Missouri‐Columbia Columbia Missouri USA; ^3^ Department of Biological Sciences Delaware State University Dover Delaware USA

**Keywords:** hc‐siRNA, miRNAs, phasiRNAs, potato, small RNAs

## Abstract

Small RNAs, including microRNAs (miRNAs), phased secondary small interfering RNAs (phasiRNA), and heterochromatic small interfering RNAs (hc‐siRNA) are an essential component of gene regulation. To establish a broad potato small RNA atlas, we constructed an expression atlas of leaves, flowers, roots, and tubers of Desiree and Eva, which are commercially important potato ( *Solanum tuberosum* ) cultivars. All small RNAs identified were observed to be conserved between both cultivars, supporting the hypothesis that small RNAs have a low evolutionary rate and are mostly conserved between lineages. However, we also found that a few miRNAs showed differential accumulation between the two potato cultivars, and that hc‐siRNAs have a tissue specific expression. We further identified dozens of reproductive and non‐reproductive phasiRNAs originating from coding and noncoding regions that appeared to exhibit tissue‐specific expression. Together, this study provides an extensive small RNA profiling of different potato tissues that might be used as a resource for future investigations.

## 1 INTRODUCTION

Potato (*Solanum tuberosum L*.) is the third largest food crop in the world (Tiwari et al., 2022). It belongs to the *Solanaceae* family and is native to the Andes region in South America, where it was first domesticated. Potato grows as an herbaceous multi‐stemmed plant and bears pinnate leaves. Flowers emerge in the summer and may display white, pink, blue, or purple color with yellow stamens. When produced, potato fruits display a green color with cherry tomato‐like berries filled with many seeds. Potatoes are cultivated for their tubers, which are enlarged, underground stems that function as storage organs for starch and other nutrients. Most cultivated potatoes are autotetraploid (2n = 4x = 48), although their ploidy can vary from diploid to pentaploid (Machida‐Hirano, 2015). There are also wild potato species that are hexaploid (Watanabe, 2015). Due to their complex genetics, potatoes are clonally propagated through tubers and through tissue culture. The potato plant is morphologically diverse, with thousands of cultivated varieties (or cultivars) and landraces. However, a much lower diversity is found in commercially produced potatoes. In this work, we focused on two cultivars, namely Desiree and Eva that were developed in the Netherlands and the United States, respectively (Figure S1). Tubers of Desiree have yellow flesh and red skin. Desiree is valued for its resistance to drought and moderate resistance to diseases (Jones & Vincent, 2018). The Eva cultivar was selected from a cross between the Stuben cultivar and bulk pollen collected from clones that were neotuberosum × tuberosum hybrids (Plaisted et al., 2001). This cultivar has white flesh and skin and is appreciated for its well‐shaped and uniform tubers. Eva is extremely resistant to diseases, including especially potato virus Y and potato virus X.

As a result of the many challenges associated with conventional breeding for potato improvement, alternative strategies are required to manage the constraints faced in the cultivation of this crop. The use of genetic engineering in potato improvement is likely to make considerable strides thanks to advances in genomics and genomics technologies. Thus, in the last decade, several potato reference genomes have been sequenced, including the doubled monoploid *Solanum tuberosum* Group phureja DM1–3 (Xu et al., 2011), the wild diploid *S. commersonii* (Aversano et al., 2015), and the diploid, inbred clone of *Solanum chacoense* ‐ M6 (Leisner et al., 2018). Given that potato is an autotetraploid, it has been suggested that its polyploid nature resulted from duplication events (Kyriakidou et al., 2020). Thus, to capture the diversity exhibited by diverse potato genomes, different sequencing technologies are fundamental, and will lead to a better understanding of the potato genome.

The availability of potato whole genome sequences has allowed parallel sequencing and identification of small RNAs (sRNAs), which are a critical part of the regulation of many cellular functions and development (Baldrich et al., 2019; Samad et al., 2017). Plant sRNAs are between 21 and 24 nt in length and, depending on their biogenesis, can be divided into three classes: microRNAs (miRNAs), phased secondary small interfering RNAs (phasiRNA), and heterochromatic small interfering RNAs (hc‐siRNA). miRNAs are 21 nt or 22 nt in size and are derived from a single strand Polymerase II (Pol II) transcript, with a characteristic hairpin folding structure. The miRNA precursor is typically processed into a mature miRNA duplex by a diverse set of proteins, including Dicer‐like 1(DCL1) (Bologna & Voinnet, 2014; Li & Yu, 2021; Rogers & Chen, 2013). PhasiRNAs on the other hand are derived from a double strand Pol II and RNA‐dependent RNA Polymerase VI (RDR6) transcript and are processed into 21‐nt or 24‐nt mature duplexes by DCL4 or DCL5. This dicing process is triggered by a 22 nt miRNA and results in consecutive phasing sRNAs (Fei et al., 2015). The third class of sRNAs, hc‐siRNAs, are derived from repetitive regions of the genome, and are typically 24 nt in size.

In this article, we characterized sRNA accumulation in leaf, root, tuber, and flower tissues of potato cultivars Desiree and Eva, which were selected to capture a maximum diversity of sRNAs from vegetative and reproductive tissues.

## 2 METHODS

### 2.1 Plant materials

Desiree and Eva potatoes used in this study were from United States seed growers. Tuber RNA was extracted directly from seed tubers received from growers. To obtain root, leaf, and flower RNA, both cultivars were grown in a room at 16 h light and 22 ± 3°C temperature. Leaf and root RNA was extracted from 6‐week‐old plants, whereas flower RNA was isolated at flowering.

### 2.2 RNA extraction

Total RNA from leaves, flowers, and roots was extracted from three biological replicates of ground plant tissue TRIreagent (Ambion), following manufacturer's recommendations. Correspondingly, total RNA from tuber was isolated using the RNeasy Mini kit (Qiagen, USA) following manufacturer's recommendations, except that using a modified tuber extraction buffer (6 M guanidine hydrochloride, 20 mM MES hydrate, 20 mM EDTA, pH 8, 10% b‐mecaptoethanol). Quality and quantity of the extracted RNA was measured by running an RNA denaturing agarose gel, and qubit fluorometer assay.

### 2.3 Annotation of potato genome

The RH genome assembly (Zhou et al., 2020) was downloaded from Spud DB (http://solanaceae.plantbiology.msu.edu/rh_potato_download.shtml). Based on this genome, repetitive regions, rRNAs, and tRNAs were annotated using computational software. Repetitive regions were annotated by RepeatMasker (http://www.repeatmasker.org), using the Viridiplantae database, whereas rRNA genes were predicted by RNAMMER (Lagesen et al., 2007), using default parameters. tRNAs genes were predicted by tRNAscan‐SE 2.0 (Chan et al., 2021), using default parameters. All the final annotations produced by these software are available in the supporting information in gtf format.

### 2.4 sRNA library preparation, sequencing, and analysis

Small RNA libraries were generated using Somagenics RealSeq®‐AC miRNA Library Kit according to the manufacturer's protocol using barcodes. Libraries were pooled and sequenced using Illumina NextSeq 550 single end sequencing at the University of Delaware DNA Sequencing & Genotyping Center (Newark, DE, USA). Sequencing adapters were removed using cutadapt (Martin, 2011), retaining all reads between 18 and 34 nt in length without undetermined bases (cutadapt ‐ a TGGAATTCTCGGGTGCCAAGG ‐ m 18 ‐ M 34 ‐ j 0 ‐‐ max‐n 0 ‐o sample.fastq). The quality of reads was assessed using FASTQC (https://www.bioinformatics.babraham.ac.uk/projects/fastqc/). Resulting reads were aligned to the RH potato genome (Zhou et al., 2020) and different features identified using bowtie2 (Langmead & Salzberg, 2013). miRNAs and phasiRNAs were identified from each cultivar separately using ShortStack version 3.8.5 (Axtell, 2013) with default parameters. We used Bedtools (Quinlan & Hall, 2010) intersect to identify predicted *PHAS* loci clusters located in coding regions, and BLAST (Altschul et al., 1990) alignment to discard *PHAS* loci clusters that contained sequences that aligned repetitive sequences and rRNA genes. miRNAs and phasiRNAs were manually curated based on their accumulation and structure. Previously annotated miRNAs for 10 species of plants were downloaded from miRbase v22 (Kozomara & Griffiths‐Jones, 2014). To identify known miRNAs, we conducted a BLAST (Altschul et al., 1990) search using the ShortStack predicted miRNA from ShortStack categories Y and N15 as a query, and the merged list of annotated miRNAs as the subject. Differential accumulation of miRNAs was done using DeSeq2 (Love et al., 2014) and raw counts resulting from Bowtie2 mapping. We set the false discovery rate (FDR) to 5%. miRNA target prediction was done using psRNATarget (Dai et al., 2018). General mapping statistics are included in Table S1.

## 3 RESULTS

### 3.1 Potato genome annotation

To understand the genomic origin of small RNAs in potato, we annotated common features, including ribosomal RNAs (rRNAs), transfer RNAs (tRNAs) and repetitive elements (Figure S2A) in the potato RH‐89 genome. For rRNAs, we obtained 9292 genes corresponding to 8003 5.8S, 663 18S, and 626 28S. We also annotated 2555 tRNAs, representing all 22 amino acids, which corresponded to 55 out of the 64 codons (Figure S2A,B). We further found that 41.73% of the genome consisted of repetitive elements, where 37.13% were retroelements and 4.29% were DNA transposons (Figure S2A,C). A vast majority of retroelements belonged to the long‐terminal repeat (LTR) Gypsy family, followed in abundance by the LTR Copia family. This is consistent with previous studies done in Solanaceae (Baldrich et al., 2021; Galindo‐González et al., 2017). While acknowledging that this analysis is likely not exhaustive, we note that due to the repetitiveness of these features, the study is sufficient to identify the sRNAs that originate from transposable elements.

### 3.2 Potato tubers have a unique small RNA profile

**FIGURE 1:**
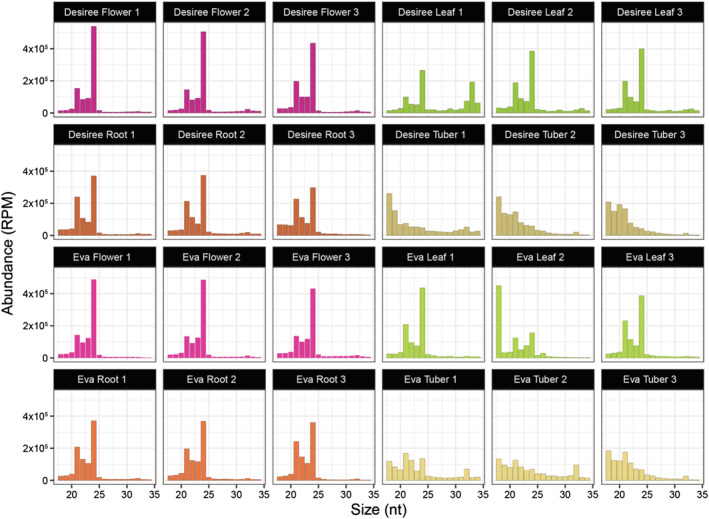
Size distribution of sRNAs mapping to the RH89 potato genome. For each of the samples, the abundance of each size class was calculated in reads per million (RPM). The *x* axis indicates the sRNA size, ranging from 18 to 34 nucleotides (nt), and the *y* axis indicates its abundance (RPM).

In this study, we prepared sRNA libraries from leaves, roots, tubers, and flowers (pooled from different stages of plant development), and independently collected three biological replicates from three different plants for each cultivar and each tissue, for a total of 24 samples. Results of sequence analysis showed that in both potato cultivars, the size distribution of sRNAs of flower, leaf, and root tissues between 18 nt and 34 nt peaked at 24 nt, with a secondary peak at 21 nt, consistent with most other plant species (Figure 1). In contrast, tubers exhibited a unique sRNA profile, displaying a more uniform size distribution with a partial peak at 21 nt (Figure 1). We observed that the 18 and 19 nt sRNAs from tubers originate from rRNAs and repetitive regions, with a small proportion originating from tRNAs (Figure S3). To determine the heterogenicity and size of each sequence, we analyzed the size distribution of distinct sRNA sequences, where each sequence is counted only once. Similar to total sRNA size distribution, we observed that flower, leaf, and root tissues displayed a predominant peak at 24 nt, usually corresponding to sRNAs from repetitive regions (Figure S4). Consistent with total sRNAs distribution recorded above, the distinct sRNAs peak at 24 nt was less predominant compared with secondary peaks at 21 and 22 nt, respectively. We also observed that the total count of distinct sRNA sequences was 10 times lower in tubers (19.4%) than in other tissues (46.4%) (Table S1).

### 3.3 microRNAs and their accumulation are conserved between Desiree and Eva potato cultivars

To date, 343 mature potato miRNAs have been reported in miRbase (Kozomara et al., 2019). We used the sRNA data generated from Desiree and Eva potato cultivars to identify known and potentially novel potato miRNAs, using the ShortStack pipeline (Axtell, 2013; Johnson et al., 2016) with default parameters and obtained 112 MIR genes in Desiree and 109 MIR genes in Eva. We merged these two outputs and obtained a total of 160 MIR genes, 62 of which were identified in both cultivars. Of these 160 miRNA precursors, 128 were already annotated in miRBase v21 (Kozomara & Griffiths‐Jones, 2014), and 32 are potential new miRNA candidates in potato. All observed miRNAs were expressed in at least one variety or tissue. Of these, 13 mature miRNAs accumulated only in Desiree and nine only in Eva (Tables S2 and S3).

**FIGURE 2:**
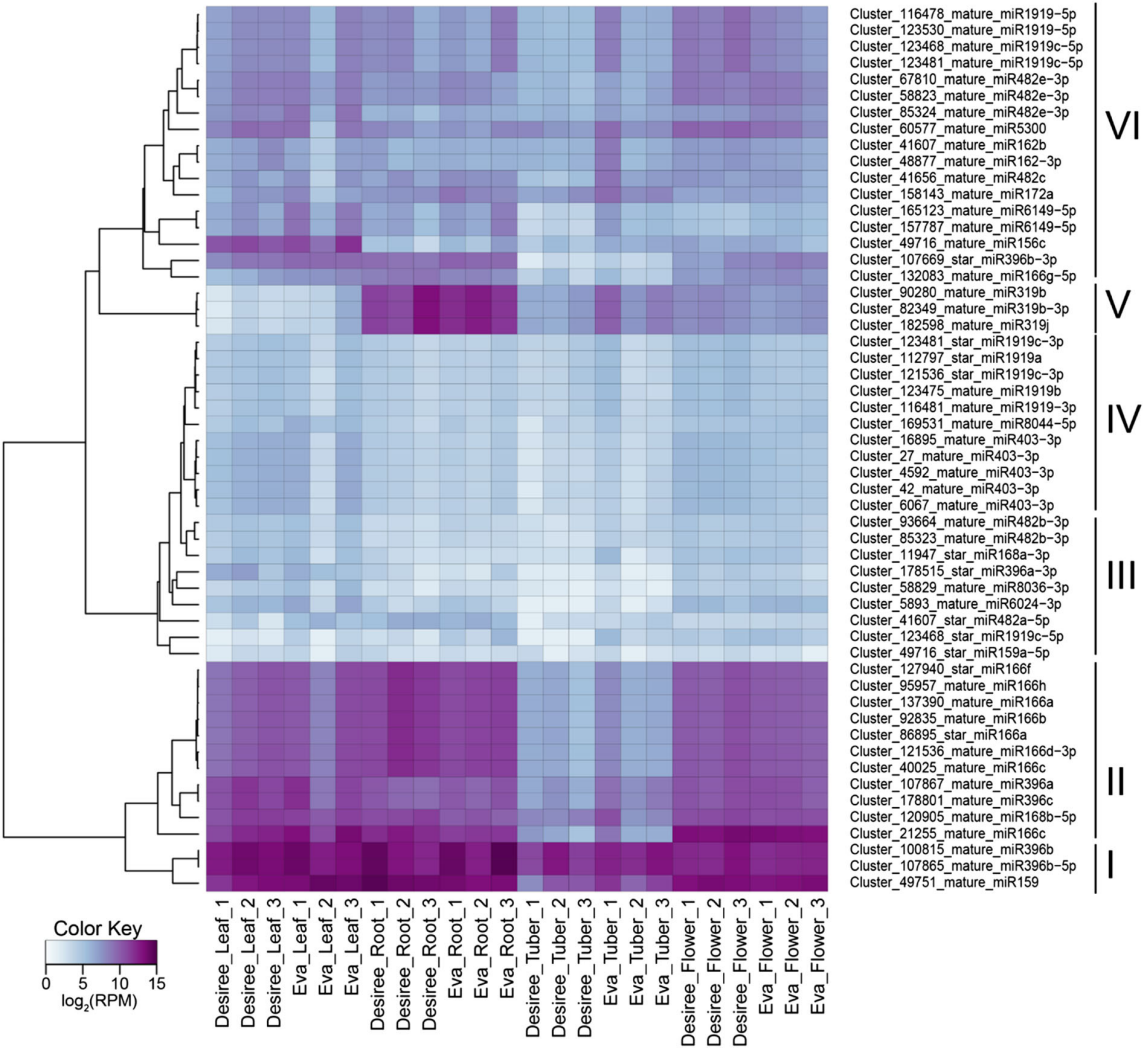
microRNAs and their accumulation are conserved between cultivars. For each sample, miRNA accumulation is represented as a heatmap, where white and light blue represent low accumulation and dark‐blue and purple represent high accumulation. We only represent annotated miRNAs that accumulate in all tissues with at least 10 reads per million (RPM). miRNAs with a similar accumulation pattern are clustered together in six distinct clusters, labeled I to VI.

To study miRNA tissue specificity, and avoid false negatives, we focused on those annotated miRNAs that accumulate in all tissues with at least 10 reads per million (RPM) (Figure 2). Three of these miRNAs were expressed in all tissues at a high level, two of which are members of the miR396 family and the other a member of the miR159 family (Cluster I). We also observed that 11 miRNAs from families miR166, miR168 and miR396 (Cluster II) accumulated in all tissues at a high level, except in tubers. Accordingly, 11 miRNAs belonging to families miR1919, miR8044 and miR403 in cluster IV accumulated at higher levels in leaves and flowers but not in roots and tubers. Finally, the three miRNAs belonging to miR319 family in cluster V accumulated at high levels in flowers, but not in the other tissues.

**FIGURE 3:**
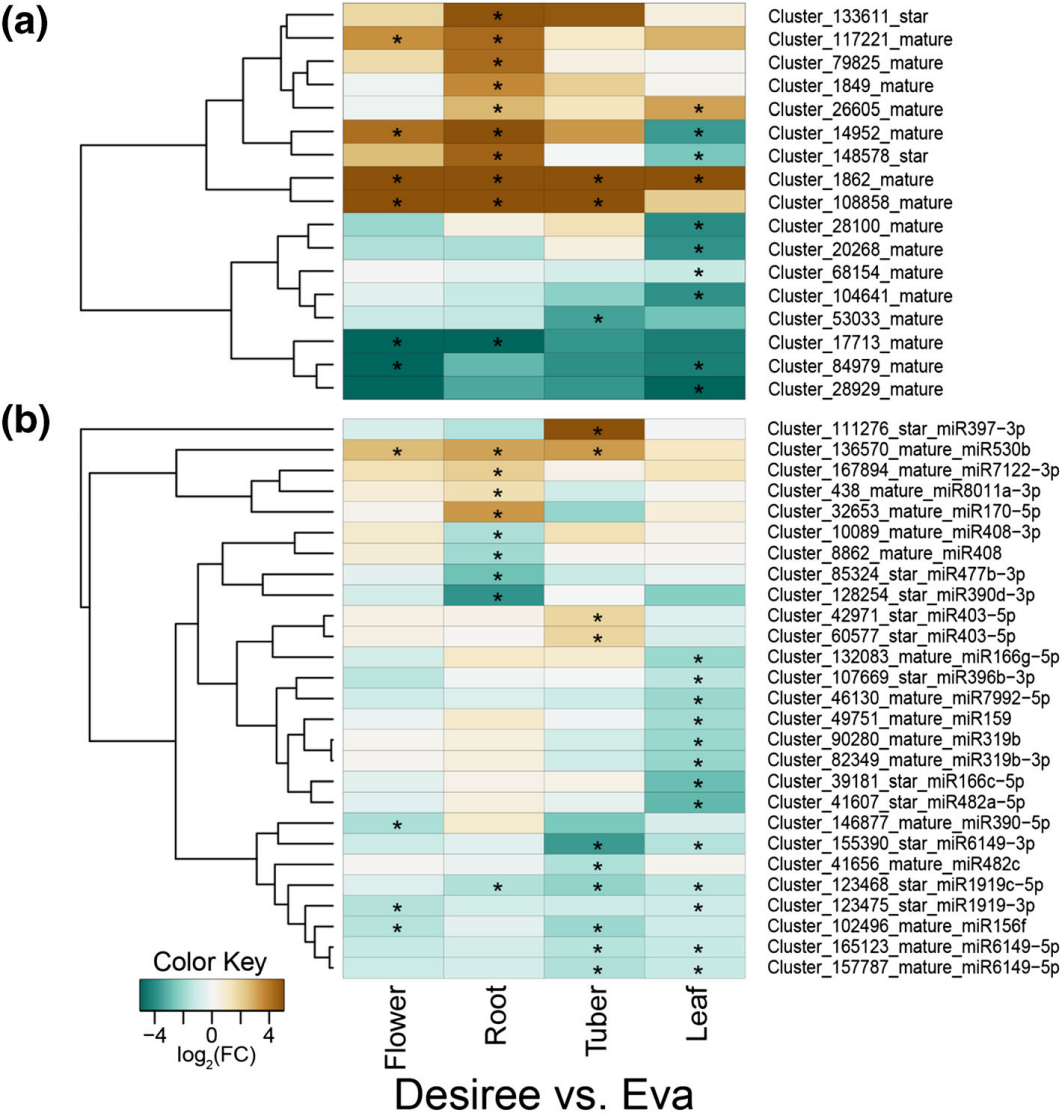
The microRNAs that have a differential accumulation between cultivars are mostly tissue specific. For each tissue, flower, root, tuber and leaf, differential miRNA accumulation is represented as a heatmap, where blue represents low accumulation in Desiree compared with Eva, and dark orange represents high accumulation in Desiree compared with Eva. The asterisks represent significant differential expression, with a *p*‐value of .05 or lower. (a) miRNAs candidates; (b) annotated miRNAs

To determine whether some of these miRNAs exhibit a difference in either of the two cultivars (Figure 3), we analyzed the differential accumulation of each miRNA in each tissue for both cultivars. A total of 44 miRNAs differentially accumulated in at least one of the tissues, 27 of which were previously annotated in miRBase and 17 potential novel miRNAs. In general, most of the differentially accumulated miRNAs showed a lower accumulation in Desiree tissues compared with Eva (blue shades in Figure 3). Notably, miRNAs in families miR6149 and miR1919 showed a lower accumulation in several Desiree tissues compared with Eva. In contrast, miRNAs from families miR403, miR530, and miR170 showed a higher accumulation in Desiree tissues than in Eva tissues (orange shades in Figure 3).

### 3.4 PhasiRNA have a similar expression in both potato cultivars

**FIGURE 4:**
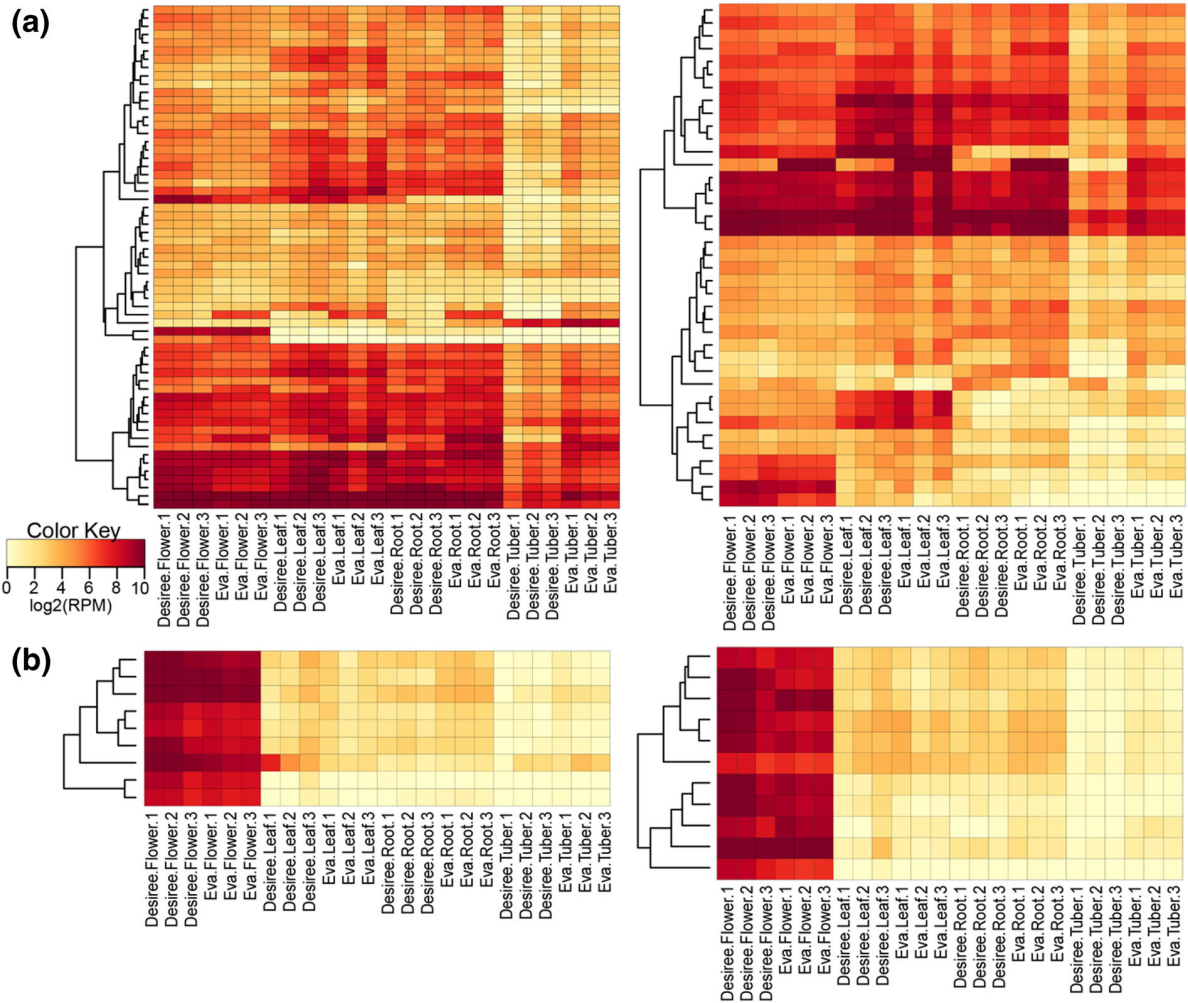
PhasiRNAs have a similar expression in both cultivars. For each sample, *PHAS* locus accumulation is represented as a heatmap, where white and light yellow represent low accumulation and red represents high accumulation. Only *PHAS* loci that are present in both cultivars in logarithmic scale of reads per million (RPM) are represented. miRNAs with a similar accumulation pattern are clustered together. (a) 21‐phasiRNA producing loci from coding (left panel) and noncoding (right panel) origin. (b) 24‐phasiRNA producing loci from coding (left panel) and noncoding (right panel) origin.

Using the same output generated by ShortStack, we identified phasiRNAs in both cultivars separately and obtained 161 21‐nt *PHAS* and 45 24‐nt *PHAS* loci in Eva, as well as 142 21‐nt *PHAS* and 75 24‐nt *PHAS* loci in Desiree. Of these, we focused on the *PHAS* loci that were common to both cultivars, corresponding to 61 21‐nt *PHAS* from coding regions, 39 21‐nt *PHAS* from noncoding regions; nine 24‐nt *PHAS* from coding regions, and 11 24‐nt *PHAS* from noncoding regions of the genome. To analyze the expression of each of these *PHAS* loci, heatmaps for all four *PHAS* categories were generated. Results showed that among the 21‐nt *PHAS* loci that overlapped with coding genes (Figure 4a), 25 were disease resistance genes, five were kinases, and one was DCL2, consistent with previous reports for other plant species (Baldrich et al., 2021; Fei et al., 2015; Reyes‐Chin‐Wo et al., 2017). We also observed a cluster of 20 loci that are highly expressed in all tissues, as well as two that appear to be flower specific (RHC10H1G1729.2 ‐ Basic chitinase and RHC08H2G1411.2 ‐ Pectinacetylesterase), one tuber specific (RHC06H2G2216.2 ‐ methyl‐CPG‐binding domain 10), and one that appears to have a higher expression in all Eva tissues (RHC04H1G0134.2 ‐ unidentified function). As for the 21‐nt *PHAS* loci from noncoding regions, four loci appear to show flower specific expression, three with leaf specific, and one with a higher expression in all Eva tissues compared with Desiree. All 24‐nt *PHAS* loci were observed to display a flower specific expression (Figure 4b and Figure S5) and showed no significant difference between Eva and Desiree.

To identify the triggers of these *PHAS* loci, we carried out a reverse analysis of target prediction using the miRNAs and *PHAS* loci identified in this study. To avoid false positives, only 22‐nt miRNAs were selected as potential triggers. This analysis identified putative triggers for 15 out of the 20 24‐nt *PHAS* loci, and 43 out of the 100 21‐nt *PHAS* loci. These triggers include miR477, miR482, miR828, miR3627, miR8036 (a member of the miR482 family), miR8050, miR4414, miR6024, miR7122, as well as two potential new miRNAs not found in miRBAse. Most of these miRNAs were previously identified in other studies as *PHAS* triggers in different species (Feng et al., 2019; Guan et al., 2014; Seo et al., 2018; Wu et al., 2017; Xia et al., 2013; Zhu et al., 2013) (Table S4).

### 3.5 hc‐siRNAs have a tissue‐specific accumulation pattern

**FIGURE 5:**
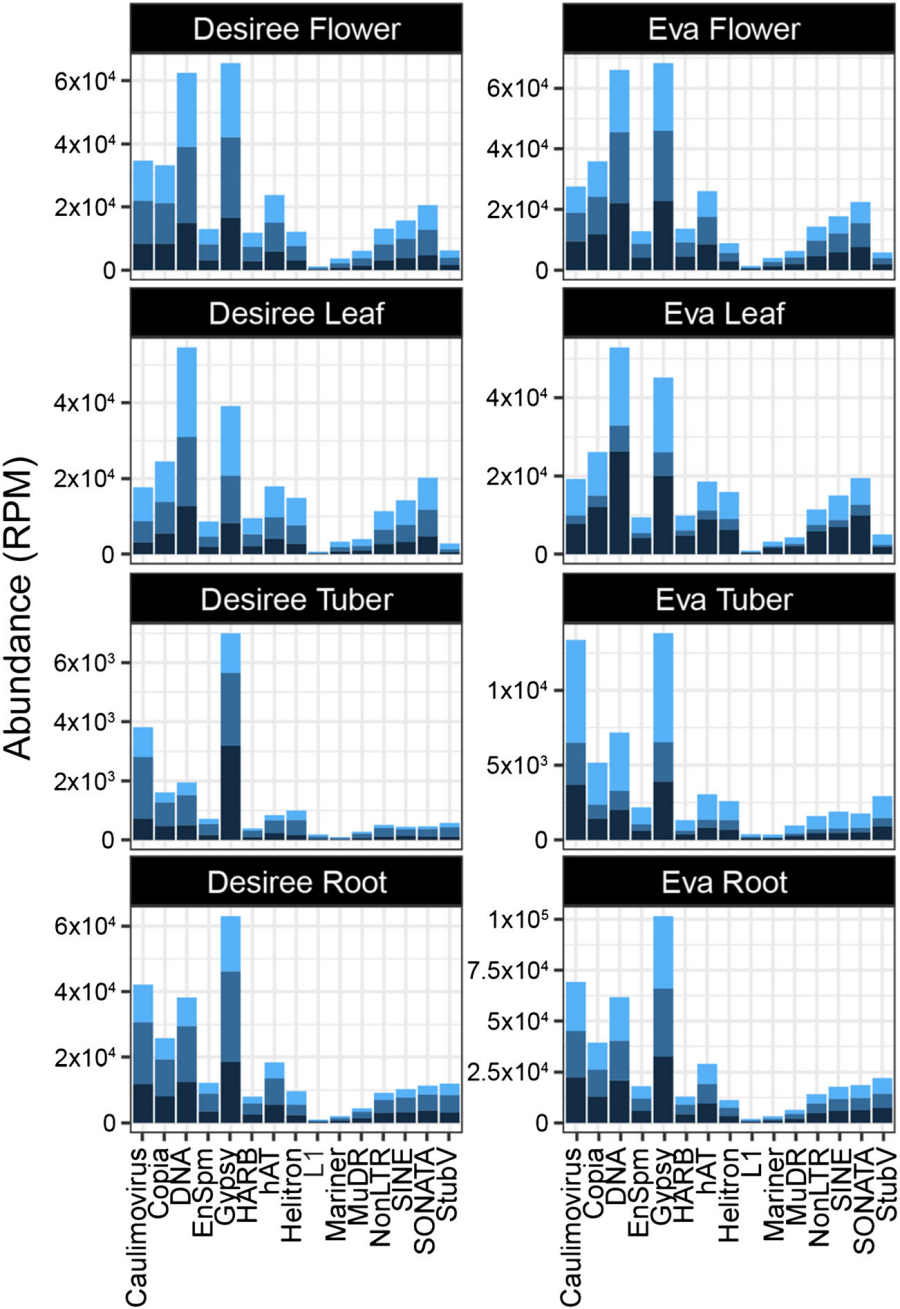
hc‐siRNAs show a tissue specific accumulation pattern. For each tissue, hc‐siRNA accumulation is represented as a bar plot, where each shade of blue represents a different biological replicate. The *x* axis indicates transposable element of origin, and the *y* axis indicates its abundance (RPM). The three shades of blue represent the three biological replicates for each sample type.

Here, we defined heterochromatic siRNAs (hc‐siRNAs) as all small RNAs reads that originate from repetitive regions of the genome, as defined by RepeatMasker. More specifically, we focus on the hc‐siRNAs that derive from transposable elements (TEs). To determine tissue accumulation of hc‐siRNAs, we first identified TEs, and then queried our data to quantify the number of sRNAs mapping each superfamily of TE (Figure 5). We observed that in both cultivars, each tissue presents a specific hc‐siRNA origin and abundance. For example, in flowers and leaves, most of the hc‐siRNAs originate from DNA and Gypsy elements, followed by Copia and endogenized caulimoviruses. However, in tubers and roots, the most abundant hc‐siRNAs originate from Gypsy and endogenized caulimoviruses. Gypsy elements were the most abundant TE annotated in the genome (~30%; Figure S3); thus, there was a correlation between the number of TE and the number of sRNAs. However, caulimovirus sequencies represented only 1.5% of the genome even though accounting for the second most abundant sRNAs. We note that sRNAs derived from endogenized viruses have been reported in potato (Geering et al., 2014); have been suggested to be involved in virus defense (Niraula & Fondong, 2021) and may be a source of novel genetic material.

A size distribution analysis of hc‐siRNAs derived from different TEs showed that reads originating from caulimovirus and StubV (a new type of endogenous florendovirus, (Geering et al., 2014) were mostly 22 nt long. In contrast, reads originating from Helitron elements were 21 nt long, whereas reads originating from all the other elements were mostly 24 nt long (Figure S6A).

## 4 DISCUSSION

In this study, we analyze sRNA datasets from leaf, flower, root, and tuber tissues of commercial potato cultivars Eva and Desiree. This analysis found that only 6% of the miRNAs identified in our samples, corresponding to 21 out of 320, are apparently cultivar specific. Additionally, these apparently cultivar‐specific miRNAs that were previously annotated in miRBase have members from the same family present that should have the same targets. This, added to the fact that the accumulation of almost all miRNAs is conserved between both cultivars, suggests that the apparent cultivar‐specific miRNAs are more an artifact than the result of a speciation. This leads us to conclude that all miRNAs that were identified in this study are conserved in both cultivars.

We also found that some miRNAs have a significantly different accumulation in both cultivars, these include miR397‐3p, miR530, miR7122, miR170, and miR403. These miRNAs, which were more abundant in Desiree compared with Eva, have been found to be involved in plant responses to stress (Dong & Pei, 2014; Kohli et al., 2014; Li et al., 2021; Manacorda et al., 2021; Xia et al., 2013). Future research efforts would determine whether these miRNAs are responsible for some of the stress responsive differences between Desiree and Eva.

In these same datasets, we identified phasiRNAs originating from coding and noncoding RNAs and producing both 21‐nt and 24‐nt siRNAs. As reported previously in other Solanaceae species (Baldrich et al., 2021; Xia et al., 2019), all the identified 24‐nt phasiRNAs from coding and noncoding genes, were found only in flowers. Furthermore, most 21‐nt phasiRNAs from coding genes were observed to be from disease resistance genes and kinases. We hypothesize that this additional layer of regulation allows for fine‐tuned expression, in which genes can be continuously transcriptionally active and regulated post‐transcriptionally at the same time, allowing for a fast response when needed.

The main role of hc‐siRNAs is to maintain genome integrity by regulating epigenetic modifications of repetitive elements. We previously reported that potato lacks hc‐siRNAs, likely due to its mostly asexual reproduction nature (Baldrich et al., 2021). However, here, we observed that all potato tissues, except for tubers, produce a typical level of hc‐siRNAs (Figure S6B). We postulate that this difference might be driven by the sampling bias of previous studies, mostly centered in tubers. To fully understand the connection between mode of reproduction and silencing pathways, future studies might be necessary to analyze hc‐siRNAs in sexually and asexually reproducing potatoes.

Here, we also identified three types of hc‐siRNAs, including 21 nt and originating from Helitrons, 22 nt and originating from integrated viral sequences, and 24 nt and originating from all other TEs. The presence of 21‐ and 22‐nt hc‐siRNAs from Helitron, caulimoviruses, and StubV, suggests that these endogenized genes might be transcriptionally active and their silencing is mediated by RDR6 and DCL2 (Fultz et al., 2015; Nuthikattu et al., 2013). In the future, it would be interesting to generate RNA‐seq data from these tissues to determine whether these TEs are transcribed. It would also be interesting to analyze the accumulation of hc‐siRNA in potato RDR and/or DCL mutants, to clarify the silencing pathway of each TE superfamily.

Together, data presented here show a complete sRNA atlas of miRNAs, phasiRNAs, and hc‐siRNAs in four different tissues of two commercial potato cultivars. Our results support the hypothesis that the vast majority of sRNAs are not cultivar specific. However, their accumulation is different and might have some physiological implications that would need to be studied in future research efforts.

## CONFLICTS OF INTEREST

The authors declare no conflicts of interest associated with the work described in this manuscript.

## AUTHOR CONTRIBUTIONS

PB, VNF and BCM designed the experiments. AL generated the libraries. PB and AL analyzed the data. PB and VNF wrote the manuscript.

## Supporting information


**Data S1.** Supporting Information

**Data S2.** Supporting Information

**Figure S1: Phenotype of Eva and Desiree potato cultivars.** A) corresponds to Desiree (plants left panel and tubers right panel) and B) corresponds to Eva (plants left panel and tubers right panel).
**Figure S2. RH89 potato genome annotation.** A) Workflow and summary of the annotation of rRNA, tRNAs, and repetitive regions of the genome. B) Summary of the codon distribution of the tRNAs identified in the RH89 genome using RNAmmer. C) Summary of the repetitive elements identified in the RH89 genome using RepeatMasker.
**Figure S3. Size distribution of sRNAs mapping different features of the RH89 potato genome**. For each of the samples, the abundance of each size class was calculated in reads per million (RPM). The x axis indicates the sRNA size, ranging from 18 to 34 nucleotides (nt), and the y axis indicates its abundance (RPM). The different colors indicate the different origins of each read.
**Figure S4. Size distribution of distinct sRNAs mapping to the RH89 potato genome**. For each of the samples, each read with the same sequence were counted only once and the abundance of each size class was calculated in reads. The x axis indicates the sRNA size, ranging from 18 to 34 nucleotides (nt), and the y axis indicates its abundance (reads).
**Figure S5: Example of a flower specific 24 nt phasiRNA**. A Desiree Flower; B Eva Flower; C Desiree leaf, root and tuber; D Eva; leaf, root, and tuber. In this viewer, individual small RNA sequences are displayed as small dots in different colors according to their sizes (unique sequences as filled dots and duplicated sequences as hollow dots). This viewer also displays genes on each strand as a series of red or blue narrow rectangles, repeat data (shadow boxes of different colors and heights), the k‐mer line plot as a purple line graph, and the sum of sequence abundances as bars.
**Figure S6. Size distribution of reads mapping to transposable elements (TE) of the RH89 potato genome**. A) For each of the samples, the abundance of each size class was calculated in reads per million (RPM). The x axis indicates the sRNA size, ranging from 18 to 34 nucleotides (nt), and the y axis indicates its abundance (RPM). B) Cumulative plot of the abundance of reads corresponding to each TE superfamily, color‐coded by sample. The x axis indicates the TE superfamily, and the y axis indicates its abundance (RPM).

**Table S1. General mapping statistics**. For each sample, this table contains: the number of total reads, the number and percentage of clean reads after the quality control and adapter trimming, the number and percentage of genome mapping reads, ribosomal RNA mapping reads, and transfer RNA mapping reads.
**Table S2.** microRNA identification. For each identified miRNA, we provide the miRNA family, the precursor length and sequence, both mature miRNAs' length and sequence, and the precursor's genome location.
**Table S3.** microRNA accumulation. For each sample, we provide the miRNA accumulation. These data that was used for differential analysis.
**Table S4.** *PHAS* loci triggers. For each identified phasiRNA, the psRNA target output for their predicted trigger. There are two categories, 21 nt and 24 nt phasiRNAs.

## Data Availability

The data discussed in this publication have been deposited in NCBI's Gene Expression Omnibus (Edgar et al., 2002) and are accessible through GEO Series accession number GSE213986 (https://www.ncbi.nlm.nih.gov/geo/query/acc.cgi?acc=GSE213986).
