# Supplementary material for: An atlas of small RNAs from potato
Source: Plant Direct. 2022 Dec 14;6(12):e466. doi: 10.1002/pld3.466 (PMC9751654; doi:10.1002/pld3.466)
Supplement: Supplementary file 3 — Figure S1: Phenotype of Eva and Desiree potato cultivars. A) corresponds to Desiree (plants left panel and tubers right panel) and B) corresponds to Eva (plants left panel and tubers right panel). Figure S2. RH89 potato genome annotation. A) Workflow and summary of the annotation of rRNA, tRNAs, and repetitive regions of the genome. B) Summary of the codon distribution of the tRNAs identified in the RH89 genome using RNAmmer. C) Summary of the repetitive elements identified in the RH89 genome using RepeatMasker. Figure S3. Size distribution of sRNAs mapping different features of the RH89 potato genome. For each of the samples, the abundance of each size class was calculated in reads per million (RPM). The x axis indicates the sRNA size, ranging from 18 to 34 nucleotides (nt), and the y axis indicates its abundance (RPM). The different colors indicate the different origins of each read. Figure S4. Size distribution of distinct sRNAs mapping to the RH89 potato genome. For each of the samples, each read with the same sequence were counted only once and the abundance of each size class was calculated in reads. The x axis indicates the sRNA size, ranging from 18 to 34 nucleotides (nt), and the y axis indicates its abundance (reads). Figure S5: Example of a flower specific 24 nt phasiRNA. A Desiree Flower; B Eva Flower; C Desiree leaf, root and tuber; D Eva; leaf, root, and tuber. In this viewer, individual small RNA sequences are displayed as small dots in different colors according to their sizes (unique sequences as filled dots and duplicated sequences as hollow dots). This viewer also displays genes on each strand as a series of red or blue narrow rectangles, repeat data (shadow boxes of different colors and heights), the k‐mer line plot as a purple line graph, and the sum of sequence abundances as bars. Figure S6. Size distribution of reads mapping to transposable elements (TE) of the RH89 potato genome. A) For each of the samples, the abundance of each size class wa [file PLD3-6-e466-s004.pptx]

## Slide 1
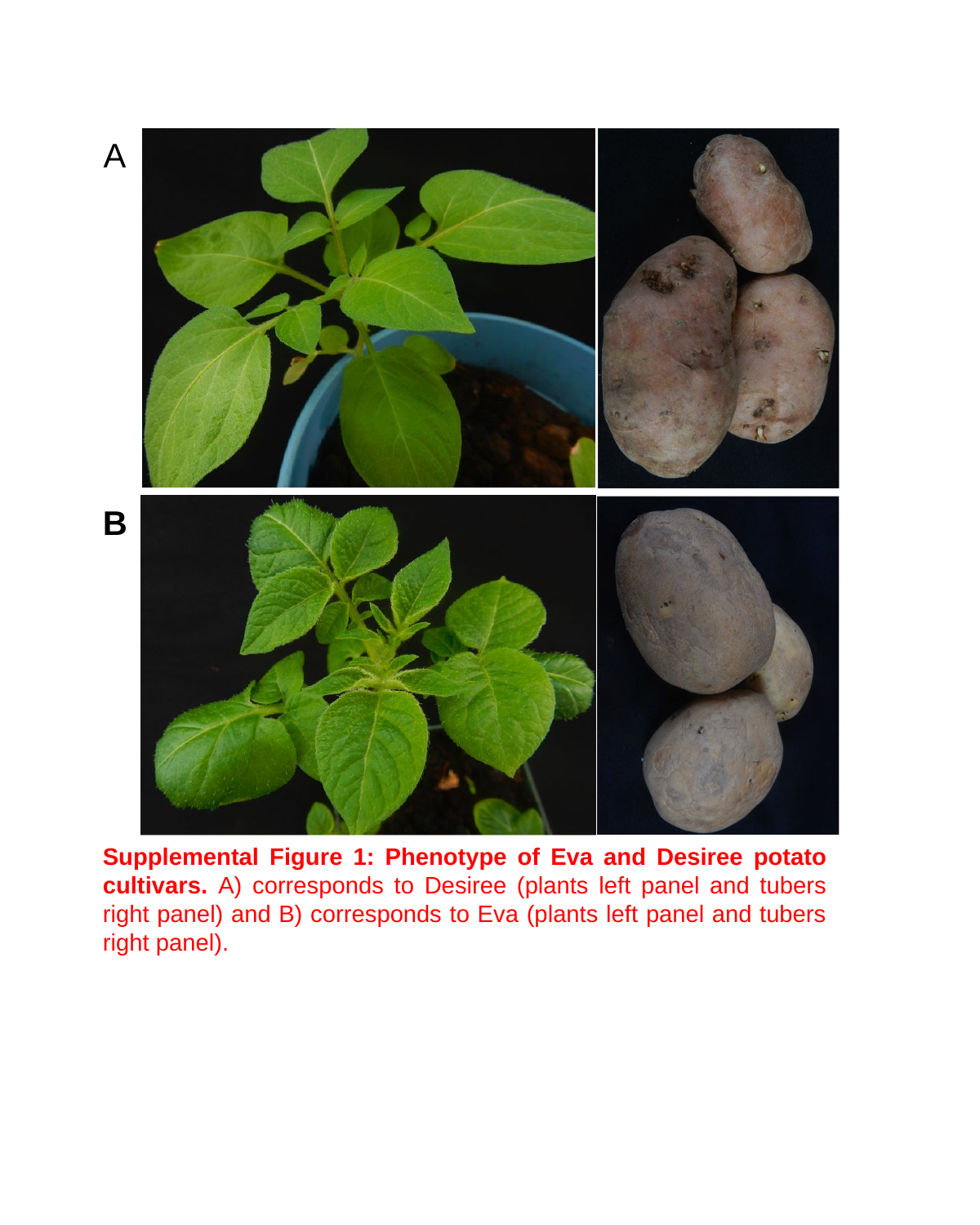

A
B
Supplemental Figure 1: Phenotype of Eva and Desiree potato cultivars. A) corresponds to Desiree (plants left panel and tubers right panel) and B) corresponds to Eva (plants left panel and tubers right panel).

## Slide 2
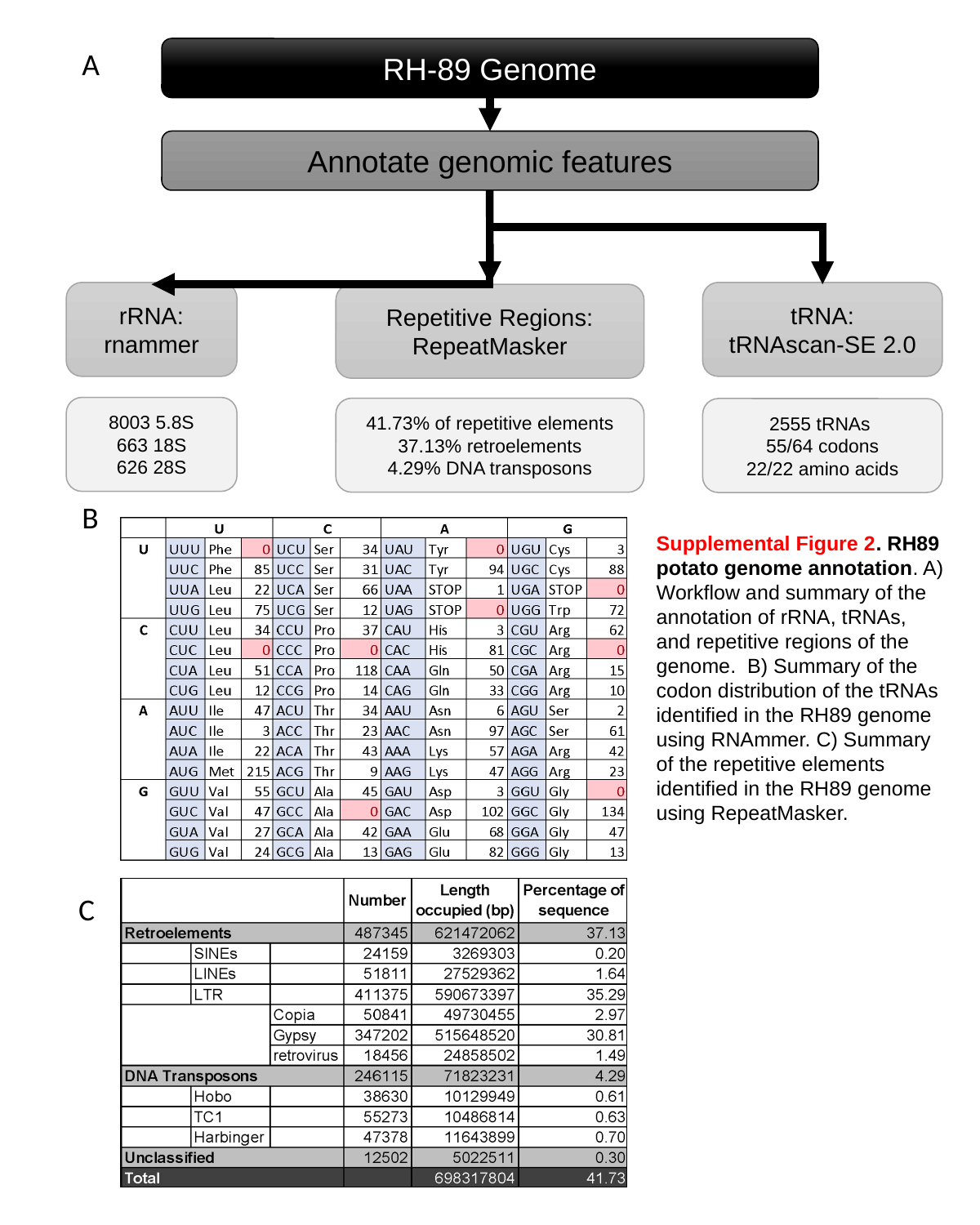

A
RH-89 Genome
Annotate genomic features
rRNA:
rnammer
tRNA:
tRNAscan-SE 2.0
Repetitive Regions:
RepeatMasker
8003 5.8S
663 18S
626 28S
41.73% of repetitive elements
37.13% retroelements
4.29% DNA transposons
2555 tRNAs
55/64 codons
22/22 amino acids
B
Supplemental Figure 2. RH89 potato genome annotation. A) Workflow and summary of the annotation of rRNA, tRNAs, and repetitive regions of the genome. B) Summary of the codon distribution of the tRNAs identified in the RH89 genome using RNAmmer. C) Summary of the repetitive elements identified in the RH89 genome using RepeatMasker.
C

## Slide 3
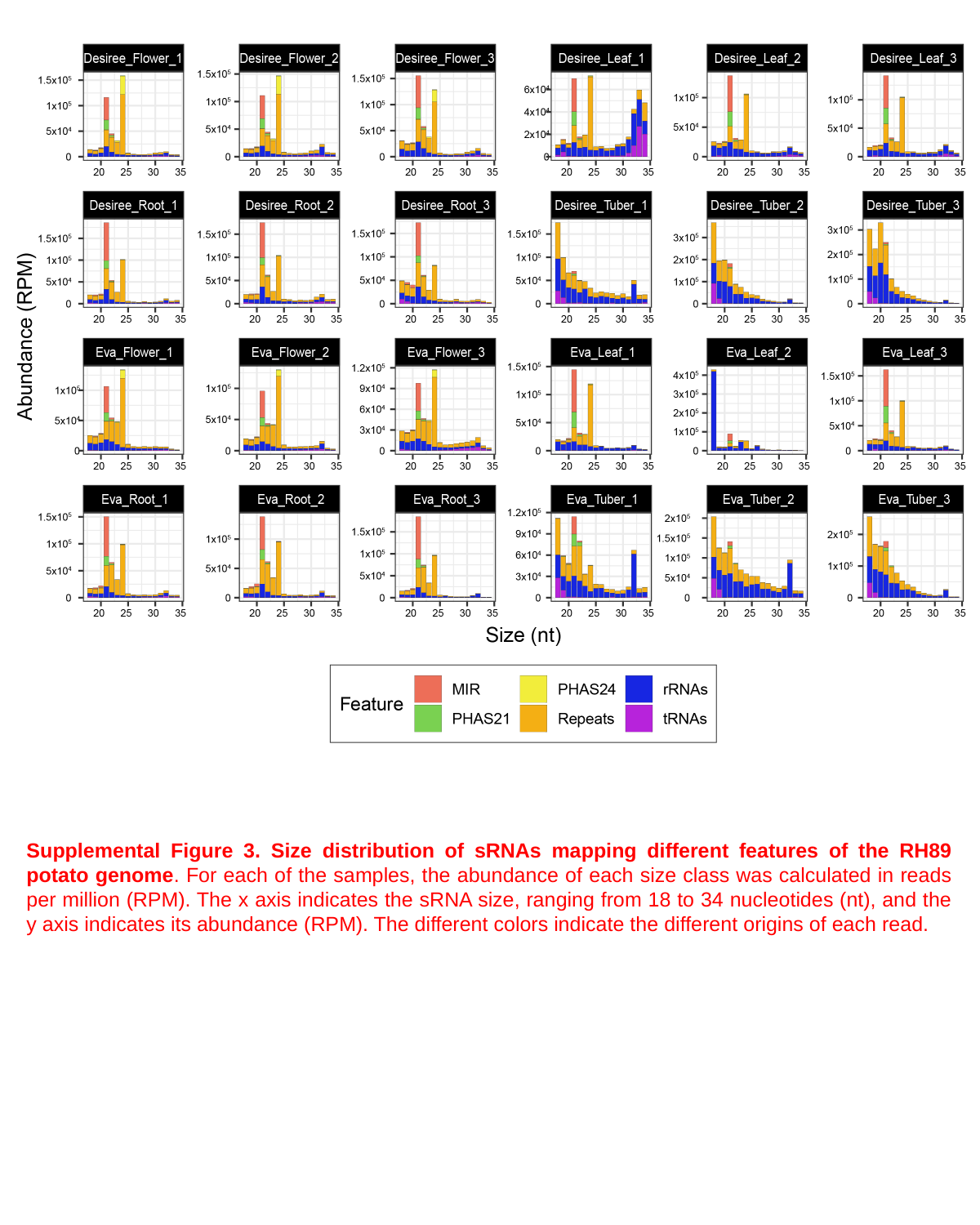

Supplemental Figure 3. Size distribution of sRNAs mapping different features of the RH89 potato genome. For each of the samples, the abundance of each size class was calculated in reads per million (RPM). The x axis indicates the sRNA size, ranging from 18 to 34 nucleotides (nt), and the y axis indicates its abundance (RPM). The different colors indicate the different origins of each read.

## Slide 4
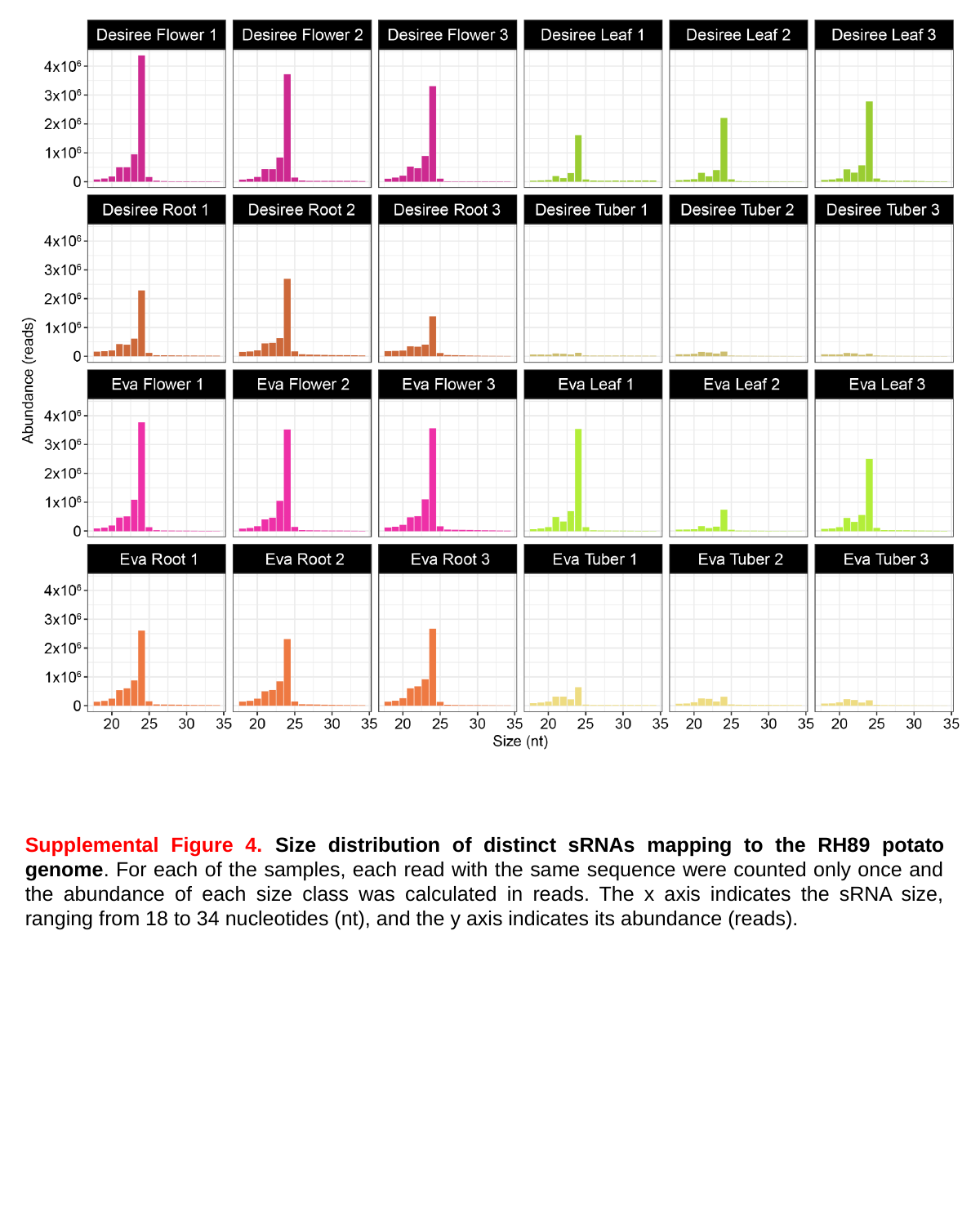

Supplemental Figure 4. Size distribution of distinct sRNAs mapping to the RH89 potato genome. For each of the samples, each read with the same sequence were counted only once and the abundance of each size class was calculated in reads. The x axis indicates the sRNA size, ranging from 18 to 34 nucleotides (nt), and the y axis indicates its abundance (reads).

## Slide 5
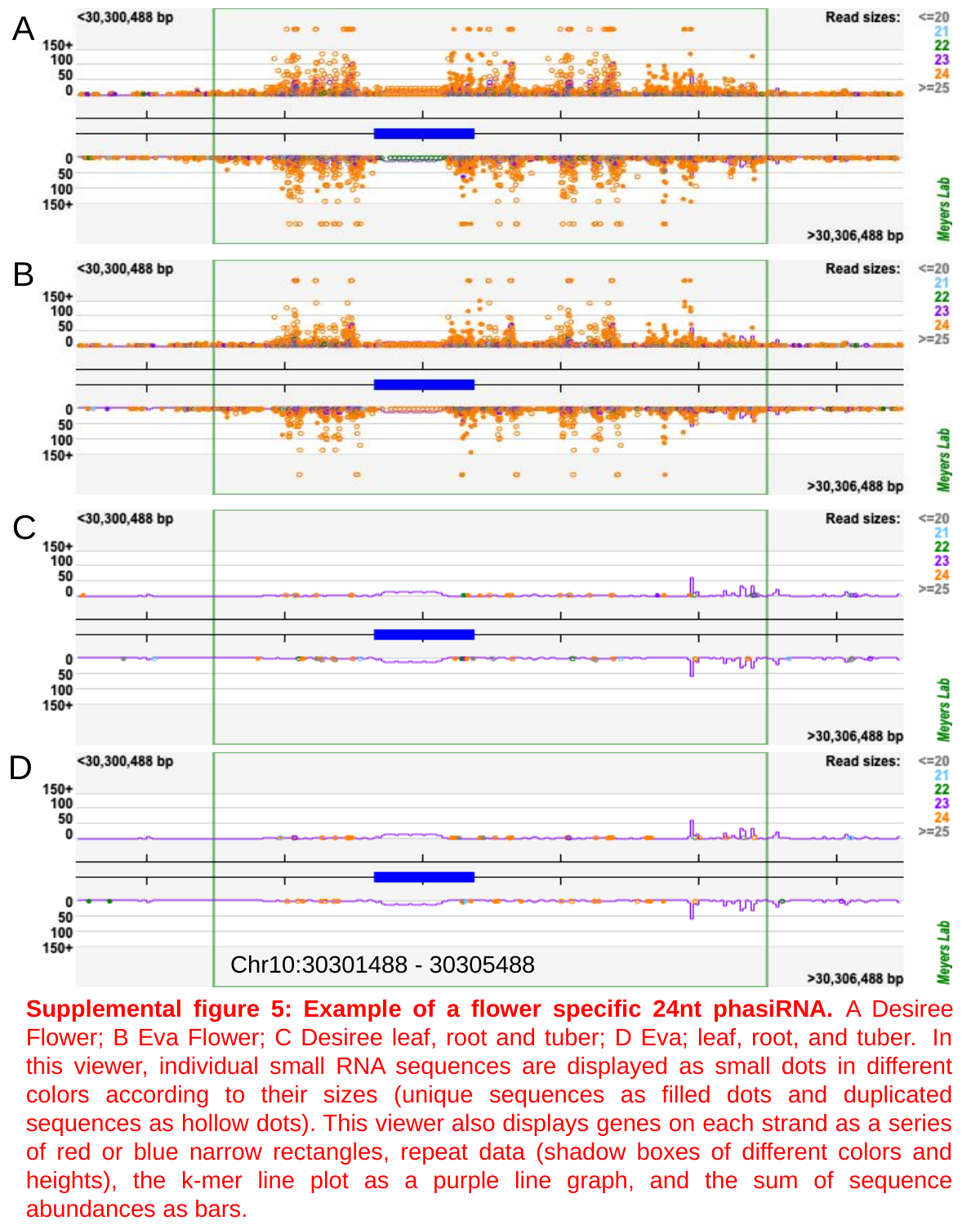

A
B
C
D
Chr10:30301488 - 30305488
Supplemental figure 5: Example of a flower specific 24nt phasiRNA. A Desiree Flower; B Eva Flower; C Desiree leaf, root and tuber; D Eva; leaf, root, and tuber.  In this viewer, individual small RNA sequences are displayed as small dots in different colors according to their sizes (unique sequences as filled dots and duplicated sequences as hollow dots). This viewer also displays genes on each strand as a series of red or blue narrow rectangles, repeat data (shadow boxes of different colors and heights), the k-mer line plot as a purple line graph, and the sum of sequence abundances as bars.

## Slide 6
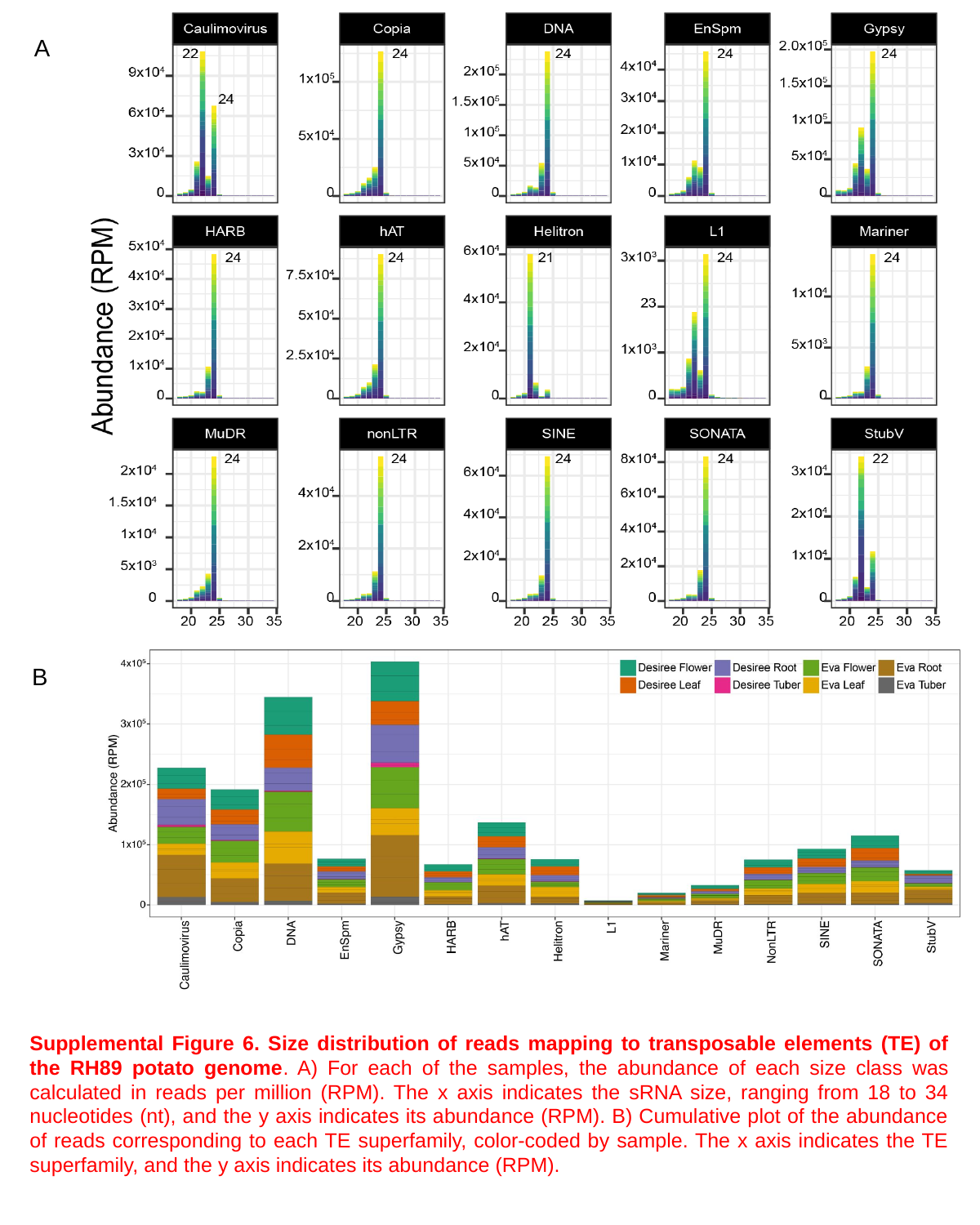

A
B
Supplemental Figure 6. Size distribution of reads mapping to transposable elements (TE) of the RH89 potato genome. A) For each of the samples, the abundance of each size class was calculated in reads per million (RPM). The x axis indicates the sRNA size, ranging from 18 to 34 nucleotides (nt), and the y axis indicates its abundance (RPM). B) Cumulative plot of the abundance of reads corresponding to each TE superfamily, color-coded by sample. The x axis indicates the TE superfamily, and the y axis indicates its abundance (RPM).
