# Supplementary material for: An atlas of small RNAs from potato
Source: Plant Direct. 2022 Dec 14;6(12):e466. doi: 10.1002/pld3.466 (PMC9751654; doi:10.1002/pld3.466)
Supplement: Supplementary file 2 — Data S2. Supporting Information [file PLD3-6-e466-s003.pdf]

Reviewer #1:

In this manuscript, Baldrich and Liu et al. comprehensively annotated and analyzed small RNAs from different tissues of two common potato cultivars. The annotation and accumulation profiles of sRNAs of different potato tissues provide useful information to the field. Overall, the experiments and analyses were well performed. I have a few concerns and suggestions for the authors to consider.
Thank you so much for taking the time to review our work.

1. The total sRNA size distribution in tuber tissues is interesting. Among all small RNAs in tuber tissues, especially in the Desiree cultivar, the 18-19 nt RNAs are highly abundant (Fig. 1). However, the size distribution of distinct sRNAs in tuber tissues still peaks at 24 and 21 nt (compare the supplemental fig. 2 to fig. 1). This suggests that the majority of the 18-19 nt RNAs in these analyses have redundant sequences. Because small RNAs in plants are usually 20-24 nt in size, I am curious whether the 18-19 nt RNAs originate from annotated small RNA loci in the potato genome. Alternatively, they can be produced from ribosome RNAs or tRNAs. To address this, the authors can filter out any RNAs that are mapped to ribosome RNA or tRNA loci and then analyze the small RNA size distribution.
We have analyzed the origin of small RNAs and color coded the reads by their genomic origin. As you can see in Supplemental Figure 3, most of the 18 and 19 nt sRNAs from tuber tissues originate from rRNAs and regions classified by RepeatMasker as repeats. This information has been added to the manuscript.

2. This manuscript showed that 24 nt phasiRNAs and some 21 nt phasiRNAs display tissue-specific expression profiles (Fig. 4). Do the trigger miRNAs and these tissue-specific phasiRNAs show correlation in expression? This could provide indirect evidence supporting the accuracy of trigger miRNA prediction.
Thank you, this is a cool idea! As a proof of concept, we checked the expression of the putative miRNA trigger and the 24PHAS loci, which are flower specific. Unfortunately, we saw that there is no correlation between the trigger and the phasiRNA precursors.

3. The term 'heterochromatic siRNAs (hc-siRNAs)' is a little confusing. Though the term hc-siRNAs is frequently used in the plant sRNA field, its definition of it is unclear. In this current manuscript, the 'hc-siRNAs' are indeed TE-derived sRNAs. Whether or not they originate from heterochromatin is not discussed in the manuscript.
We agree that there is a lot of jargon in the small RNA field, and that not all small RNAs in plant genomes can be uniquely classified. And thank you for pointing out the information missing about the connection between heterochromatin and 24-nt siRNAs. We have added some details to the manuscript, and now define hc-siRNAs as small RNAs derived from repetitive regions identified by RepeatMasker.

4. Description for supplemental Fig.3B is missing in the figure caption; Please include a figure legend to explain different colors in Supplemental Fig. 3A.
Thank you, we have added the description. Now it is Supplemental Figure 4.

5. What does the asterisk mean in Fig. 3?
Thank you, we have added the meaning to the figure legend.

6. Please specify the false discovery rate (FDR) in the differential gene expression analysis.
Thank you. We have added this information in the methods section.

7. In line 69, Baldrich et al. (2019) paper is not listed in the reference. In line 179, Kozomara et al. (2019) is not listed in the reference. Please double-check.
Thank you, we added these references to the list.

Reviewer #2:

In this manuscript, Baldrich and colleagues analyze the sRNA populations of two potato cultivars, Desiree and Eva. The authors found relatively small changes in the sRNA populations, with only some miRNAs and hc-siRNAs showing potentially interesting changes. The manuscript is focused on the analysis of sRNAs and lacks any degradome or transcriptomics data that could have helped to understand the function of the identified sRNA differences. Nevertheless, in my opinion, this is still an important resource for the sRNA community, since it identifies differences in the accumulation of sRNAs between different tissues, a feature that has not been explored in depth for many cultivated

species. The paper is concisely written and contains appropriate figures. I have some comments that I hope could help to understand in detail the described changes.
Thank you so much for taking the time to review our work.

Major comments:

1. For a manuscript that describes the overall population of sRNAs between different tissues and cultivars, I am missing a more in detail description of the different sRNA profiles for each of the categories described in the paper: coding genes, TEs, tRNAs, rRNAs. How do these different populations change between tissues? How do they change between cultivars? How do the sRNA size profiles for each category look like?
Thank you for your comment. We have added a figure that shows the different size distribution for the different categories and for each sample; this is Supplemental Figure 2.

2. I have a similar comment regarding miRNA accumulation. It would be interesting if the authors could provide a miRNA size profile analysis for the different tissues and cultivars analyzed here. Since miRNAs of different sized can have different roles, this is especially important for this sRNA category.
This is now included in Supplemental Figure 2. However, we did not see any significant differences between samples, the vast majority of reads mapping to *MIR* loci are 21 nt in size.

3. Figure 5 is missing a color key, what makes it impossible to fully understand.
Thank you. The three shades of blue represent the three biological replicates for each sample type. We have added this information to the figure legend.

4. The authors might include a comment in the discussion regarding how the results could be influenced by the lack of a genome sequence for these particular cultivars. Since this information is missing, it is plausible that the sRNA profiles here could be different if a complete genome sequence was available.
Thank you for your comment. We do not think that a different genome might change our results significantly. Most of the *MIR* genes and TEs are highly conserved between cultivars.

5. Could the authors expand on some of the differences between the phasiRNA accumulation between the two cultivars? The paper will certainly gain in depth if an additional extra panel would be added with a more in detail analysis of an example (or two) of phasiRNAs differential accumulation in any tissue between the two cultivars.
Thank you for your suggestion. We added an example of 24nt phasiRNA expression in the different cultivars and tissues in supplemental figure 5.

6. The discussion of the manuscript might be too superficial in the analysis of the results. Could the authors expand the discussion of their results with specific examples of targets of the miRNAs or phasiRNAs identified and correlate that with the characteristics of the two cultivars analyzed here? In the introduction the authors refer to tuber flesh/skin color and disease resistance differences between the two cultivars, are the differences in miRNAs, phasiRNAs or hc-siRNAs driving or contributing to any of those differences? Alternatively, if word number restriction is the reason for that lack of depth, would a summary figure help to understand the results better?
Thank you so much for your comments. We feel that any relationship that we could establish between small RNAs and their putative targets might be too speculative. We did not generate any PARE or RNAseq data, and therefore we do not have any experimental proof to support any small RNA target. We hope to address these concerns in future work.

Minor comments:

1. In Supplementary Table 3, column B needs to be fixed, since starting at line 254, there is no miRNA family associated with the sRNA cluster. Is this intentional?
   Thank you. Yes, this was intentional, but we understand that it may be unclear. We have changed these columns to read “not identified”.

2. Supplementary Figure 1 panels B and C are missing a border on the right side.
Thank you, we have added the missing borders.

4. Is the sRNA data publicly available? That would certainly help to make this a helpful resource for the potato community.
Yes, we agree. The data are already on GEO, and they will be made available as soon as the paper is published. We are sorry that we forgot to add this information to the manuscript.

5. A similar comment might be said regarding the genomic features identified in this manuscript. It would be helpful if they were available for the community.
Yes, we will make this data available through our webpages at https://wuji.ddpsc.org/staff_resources/next-gen-websites/

6. It would be interesting/helpful if the phenotypic differences between both potato cultivars were shown with a couple of pictures in a supplementary figure.
Thank you for the suggestion. We introduced a new Supplemental Figure 1 with pictures of both cultivars, from the plant and the tubers.

7. Supplementary tables would benefit from an expanded figure legend that explains in detail the data shown in the tables.
Thank you for the comments, we have added more details to the legends.
